# The intra-day and inter-day reliability of a 6-second Wingate to determine maximal peak power in endurance-trained athletes

**DOI:** 10.1371/journal.pone.0307325

**Published:** 2024-09-06

**Authors:** Joao Henrique Falk Neto, Normand Boulé, Kelvin E. Jones, Aidan K. Comeau, Michael D. Kennedy

**Affiliations:** 1 Faculty of Kinesiology, Sport and Recreation, University of Alberta, Edmonton, Alberta, Canada; 2 Alberta Diabetes Institute, University of Alberta, Edmonton, Canada; 3 Neuroscience and Mental Health Institute, Edmonton, Alberta, Canada; Universidad de León Facultad de la Ciencias de la Actividad Física y el Deporte: Universidad de Leon Facultad de la Ciencias de la Actividad Fisica y el Deporte, SPAIN

## Abstract

Determining an athlete’s maximal peak power (MPP) is crucial in profiling endurance sports participants. While short (3 to 6 seconds) all-out efforts have been validated for MPP assessment, prior studies mainly involved non-endurance trained athletes. This study aimed to assess the intra- and inter-day reliability of a 6-second Wingate test for MPP determination in endurance athletes. Endurance-trained participants (22 males, 5 females) completed nine 6-second Wingate tests over four days (3 trials at baseline, 2 trials on each subsequent day). Analysis revealed no systematic differences in MPP (F(4.09, 106.3) = 1.88, p = 0.117) or time to peak power (χ^2^ (8) = 5.23, p = 0.732) across the trials. Reliability, assessed through the intraclass correlation coefficient (ICC) and standard error of measurement (SEM), was excellent across all trials (ICC = 0.95, SEM = 40.0W, SEM% = 3.7%). Absolute reliability improved when selecting the average or the best MPP values from each day (SEM% = 2.7% and 2.9%, respectively). Within-day reliability was consistently rated as excellent, with the best values on the 4^th^ day of tests. No significant differences in MPP values occurred between the first and second 6-second Wingate tests on days 1 to 3, with both trials demonstrating similar reliability values (SEM%: 3.2% vs 2.8%, for the first and second trials, respectively). The test also demonstrated a good sensitivity to detect a meaningful change in MPP values. In conclusion, the 6-second Wingate test proves reliable for determining MPP in endurance-trained athletes. Two trials are recommended on the first day of testing, with a single MPP likely sufficient to determine the athlete’s MPP on subsequent days.

## Introduction

Endurance exercise performance is determined by a range of characteristics, with the importance of each varying based on the event [1,2]. Events characterized by a variable pacing profile, such as road cycling, mountain biking, and cross-country skiing, for example, require athletes to alternate periods of low intensity exercise with short bouts at maximal and / or supramaximal intensities [3,4]. The supramaximal efforts performed in these events occur in what is termed the anaerobic power reserve (APR). The APR is the range of intensities that span from the maximal aerobic power (MAP) to the athlete’s maximal peak power (MPP) [2,5]. The APR has been shown to be an important concept in the prescription of interval training [6] and in explaining the ability to perform continuous [7] and intermittent [8] exercise at supramaximal intensities. The APR has also been utilized to determine an athlete’s “locomotor profile” [2], a factor that might be relevant to individualize training programs. Further, many endurance events, such as road cycling, cross country skiing, and mountain biking, also require the athletes to sprint to the finish line, an effort that occurs at a high percentage of their APR [4,9–11]. In addition to its role in determining an athlete’s APR (and locomotor profile) [2], the MPP is an important characteristic associated with performance in different endurance events. In mountain biking, for example, a high MPP is a key factor for performance, with athletes showing a 15% increase in MPP across a 10-year period [4].

While tests to determine an athlete’s MAP are commonly employed, less attention has been given to methods to determine MPP in trained endurance athletes. The 30-second Wingate test has been the most employed test to date to determine an individual’s MPP [12–14]. However, the test is considered very strenuous, and as such, has limited practical applicability [15–17].

Recent studies have validated a 6-second all-out test as a measure of MPP [13,14,18] as the MPP from the test shows a strong correlation with the values obtained from a traditional Wingate test [14]. The test also has a strong within- [13,19] and between-day reliability [17,18,20], and might provide a better estimate of the individual’s MPP, as the traditional 30-second Wingate test might underestimate the participant’s true MPP when compared to a 6-second all-out test [14,18].

To date, numerous studies have utilized a 6-second all-out effort to determine MPP. However, variations in protocols, equipment, and participant’s background yield conflicting evidence [13,17,18,20]. The need for familiarization trials and the optimal number of testing sessions to establish an athlete’s MPP remain underdetermined [13,20].

Even though a 6-second all-out test is commonly utilized to determine MPP in endurance athletes [4,5,21], only one study [19] has assessed the reliability of a 6-second all-out test in trained cyclists. The study demonstrated that the test had excellent (coefficient of variation (CV) of 2.19%) within-day reliability but did not assess the reliability of the test between days. A previous study established that cycle-trained individuals can produce stable MPP values across days when performing 3–4-second sprints, with no familiarization trial required [22]. However, the authors did not report any reliability data. In addition, these two studies utilized a fixed cadence [19] or a static start [22]. While both methods have been linked to better reliability of the test [23], previous studies that have utilized a 6-second effort to determine MPP in endurance athletes have asked participants to perform a maximal acceleration from a “flying” start [5,21]. Therefore, determining the reliability of a 6-second MPP test with a flying start in trained endurance athletes, understanding if a familiarization trial is needed, and whether multiple trials are required to establish an athlete’s MPP, is key to provide coaches, researchers, and sport scientists with best practice guidelines for determination of MPP in this cohort.

The aim of this study was to determine the intra- and inter-day reliability of a “flying” 6-second Wingate test to determine MPP in a group of trained endurance athletes. The test was chosen due to its popularity in research and applied settings [15,18], strong reliability reported in non-trained individuals [18], and the possibility of being performed with a flying start, mimicking other all-out tests employed in research settings with endurance athletes [5,21]. It was hypothesized that a 6-second Wingate test would be a reliable method to determine MPP in this cohort of athletes, with no differences across trials and no evident learning effect. Lastly, it was hypothesized that the second 6-second Wingate test on each day would yield higher and more reliable MPP values when compared to the first Wingate test.

## Methods

### Participants

The study recruited 27 trained endurance athletes. To be eligible, participants were required to be age group athletes competing at a regional, national, or international level in cycling and triathlon, have 3+ years of training experience, and to be currently training more than 4 hours per week. Based on these characteristics, participants were considered tier 2 endurance athletes [24]. The participants included 22 men (age: 33.7 ± 10.4 years, body weight: 82.1 ± 12kg) and 5 women (age: 38.4 ± 5.5 years, body weight: 64.9 ± 5.8kg). All tests occurred between January 10^th^ and August 22^nd^, 2022. The participants were informed of the procedures of the study prior to providing written informed consent to participate. The study was approved by the University of Alberta Ethics Committee.

### Procedures

**Fig 1:**
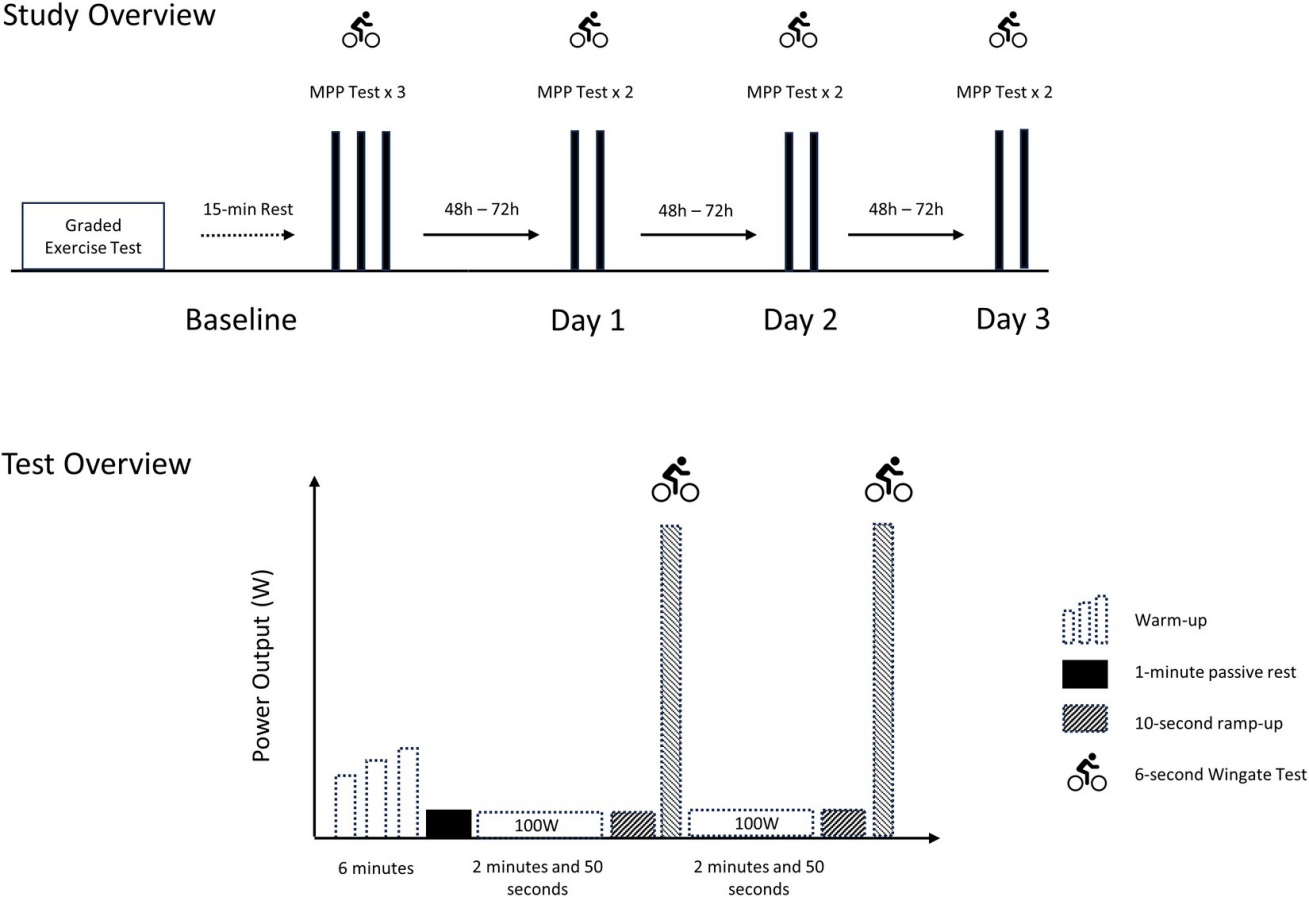
Overview of the study’s protocol.

An overview of the study’s protocol is presented on Fig 1. The participants visited the lab on 4 different occasions. During the first testing day (baseline), the participants completed three 6-second Wingate tests to determine their MPP. The participants then performed two 6-second Wingate tests on three subsequent testing days. Each participant completed a total of nine 6-second Wingate tests. The baseline tests were performed 15 minutes after the participants had completed a maximal graded exercise cycling test. All other tests were performed at the beginning of the testing session, with no prior exercise completed.

Every participant completed all tests at the same time of the day (± 2 hours), under similar temperature and humidity conditions (23.2 ± 0.2°C, 39.2 ± 12.4%). Each testing day was separated by at least 48 hours of rest. The participants were instructed to avoid strenuous exercise on the two days preceding the test, to maintain a similar sleeping routine prior to each testing day, and to avoid caffeine and alcohol in the 12 and 24 hours prior to the test, respectively. The participants were also instructed to ensure that they consumed a similar light meal 2 to 4 hours before each test. All the participants verbally confirmed that they were able to follow the researchers’ instructions.

### Testing protocol

All tests were performed on the same electronically braked cycling ergometer (Velotron Racermate, Seattle, WA). The reliability of the Velotron for determination of MPP during a 30-second Wingate test has been previously established [25]. During the first day of testing, the participants were instructed to adjust the cycling ergometer (seat height, bar position) to their preference. This setup was then recorded and replicated on each subsequent trial. Prior to starting the 6-second Wingate test, the participants performed a 6-minute warm up pedalling at 110W, with short (6 seconds) accelerations against a load equivalent to 70%, 75%, and 80% of their maximal aerobic power (MAP) at 2, 4, and 6 minutes. Following completion of the warm-up, participants rested passively for one minute. The test started with the participants pedalling at a low intensity (110W) for 2 minutes and 50 seconds. At the end of this period, the participants were asked to gradually increase their cycling cadence, aiming for a maximal, “flying” 6-second sprint at the 3-minute mark. The participants were provided with an audible 5-second countdown and the cycling ergometer software automatically applied the load at the end of this period. The participants were instructed to aim for maximal cadence at the moment that the load was applied and to continue pedalling all-out until the 6 seconds were completed. Verbal encouragement was provided on every test. The 6-second sprints were performed against a load equivalent to 7.5% of the participant’s bodyweight [18,25]. Once the first 6-second effort was completed, the participants returned to low intensity cycling (110W) for the next 2 minutes and 50 seconds, prior to completing the second all-out effort. The Velotron Software records power output every 0.1 second. MPP was determined as the highest mechanical power output recorded during the test according to the software’s instructions.

Considering the protocol, the participant’s cadence momentarily increased at the onset of the test prior to presenting a steady decline throughout the 6 seconds. Thus, a test was considered not valid if the cadence at the end of the test was the same or higher than at the start. In these cases, the data from the test was replaced with the average MPP value of all the tests from that participant.

### Statistical analysis

Statistical analysis was performed using SPSS for Windows (SPSS 26.0, Chicago, IL). Data are presented as mean and standard deviation. Statistical significance was set at p < 0.05, and when applicable, 95% confidence intervals are presented.

To determine if there were differences in MPP values obtained from the nine 6-second Wingate trials across days, a one-way repeated measures ANOVA, with a Bonferroni correction for multiple comparisons was performed. The same procedure was used to compare the best MPP values on each day and the average MPP values from each day. The assumption of normality was violated for time to peak power, and as such, Friedman’s test was utilized to determine if there were differences in time to peak power across all trials. Between-day reliability was assessed for all nine trials and based on the average and the best MPP values on each day. To determine if the between-day reliability improved throughout the study, the changes in reliability across the study (all tests, days 1 to 3, and days 2 and day 3) were analyzed. The between-day reliability of the first and second 6-second Wingate tests on days 1, 2, and 3 was also compared to determine if the second MPP trials show better reliability values than the first one. A paired-samples t-test was performed to determine if there is a difference between the mean MPP values from the first and second trials on days 1 to 3. On each testing day, the within-day reliability of the test was also determined.

Relative reliability of the 6-second Wingate test was determined via intraclass correlation coefficients (ICC _2,1_) [26,27]. The values were considered as poor (ICC < 0.5), moderate (0.5 ≥ ICC < 0.75), good (0.75 ≥ ICC < 0.90), and excellent (ICC ≥ 0.90) [28]. Absolute reliability was assessed by calculating the standard error of measurement (SEM) (SEM = SD x √ (1 ‐ ICC)) and the percentage SEM values (SEM%) [27,29]. For the SEM, 95% confidence intervals were also calculated. The smallest worthwhile change (SWC) was utilized to analyze the test’s sensitivity to detect a meaningful change and was calculated based on Cohen’s *d* effect size and the between participants standard deviation (SWC = 0.2 x SD). The test is considered to have “good” sensitivity when the SEM is smaller than the SWC, “satisfactory” sensitivity when the SEM and the SWC are the same, and “marginal” sensitivity when the SEM is larger than the SWC [15].

## Results

Only 8 (approximately 3%) out of 243 tests were considered not valid. Of these tests, four occurred in the first day of tests (baseline), with three being the first trial of that day. Five participants had tests that were considered not valid, with three participants failing one test, one participant failing two tests (in different days), and another participant having three failed tests (two in the same day, baseline).

### Intra-day reliability

**Fig 2:**
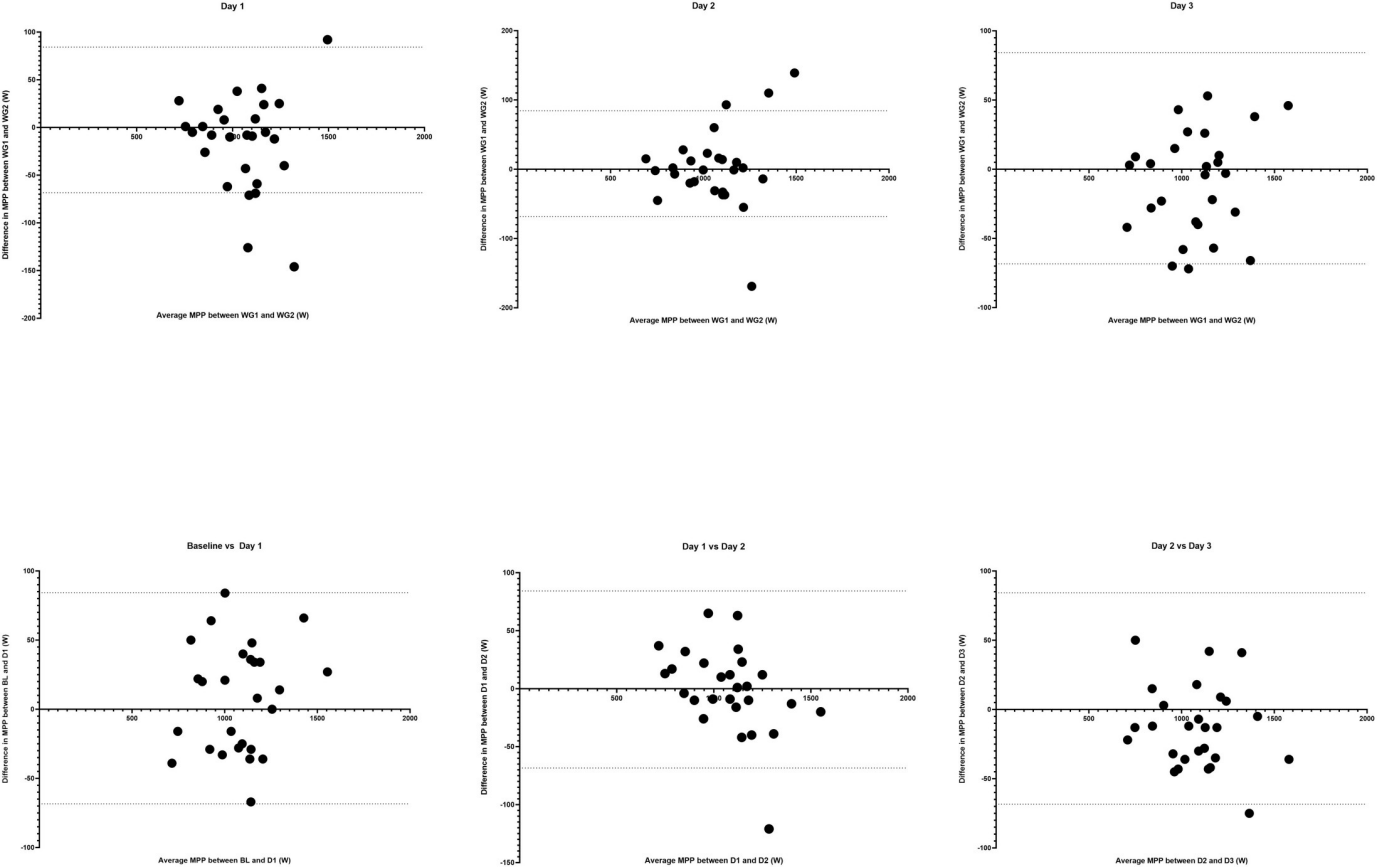
Bland-Altman plots for pairs of tests. Intra-day comparisons between the first and second 6-second Wingate tests on day 1 (top left), day 2 (top center), and day 3 (top right)**.** Inter day comparisons for the best MPP between baseline and day 1 (bottom left), day 1 and day 2 (bottom center), and day 2 and day 3 (bottom right). Dotted lines represent 95% confidence intervals. MPP: Maximal peak power, WG1: 1^st^ 6-second Wingate test, WG2: 2^nd^ 6-second Wingate test, BL: Baseline, D1: Day 1, D2: Day 2, D3: Day 3.

The within-day reliability statistics are presented in Table 1. There were no differences in MPP values across the trials on any of the testing days (p = 0.33, 0.12, 0.85, and 0.17, from baseline to day 3, respectively). The ICC values for baseline, day 1, and day 2 were 0.95, 0.96, and 0.95, respectively, while SEM% values were 3.7%, 3.3%, and 3.6% for each day. Within day reliability presented the strongest values on Day 3 (ICC = 0.98, SEM% = 2.4%). Bland-Altman plots for the first and second 6-second Wingate tests on days 1 to 3 are presented on Fig 2. During baseline testing, the SEM (39.8W) was larger than the SWC (35.9W), while on day 2 both values were the same (SEM and SWC = 38.75W). On days 1 and 3, the SEM was smaller (35.6W on day 1, and 26.2W on day 3) than the SWC (36.1W and 41.5W for days 1 and 3, respectively).

**Table 1:** Intra-day reliability data for the 6-second Wingate tests.

Day	MPP Trial 1 (Watts)	MPP Trial 2 (Watts)	MPP Trial 3 (Watts)	P-value	ICC	SEM (Watts)	SEM%	SWC (Watts)
Baseline	1042.0 ± 190.5	1057.4 ± 170.4	1053.6 ± 184.8	.33	0.95 (0.90–0.97)	39.8	3.7% (3.4% - 4.2%)	35.9
Day 1	1049.0 ± 180.8	1064.3 ± 183.5	‐‐‐	.12	0.96 (0.92–0.98)	35.6	3.3% (3.1% - 3.7%)	36.1
Day 2	1058.1 ± 201.1	1056.1 ± 190.0	‐‐‐	.85	0.95 (0.90–0.98)	38.7	3.6% (3.3% - 4.0%)	38.7
Day 3	1068.2 ± 213.0	1078.3 ± 206.3	‐‐‐	.17	0.98 (0.96–0.99)	26.3	2.4% (2.3% - 2.6%)	41.5

* Significantly different than day 2 (p < .05).

Data for MPP values presented as mean ± standard deviation. Values in parentheses represent the 95% CI. MPP: Maximal peak power; ICC: Intraclass correlation coefficient; SEM: Standard error of measurement; SEM%: Standard error of measurement as a percentage; SWC: Smallest worthwhile change.

### Inter-day reliability

Across all trials, there was no difference in MPP (F(4.09, 106.3) = 1.88, p = 0.117) or time to peak power (χ^2^ (8) = 5.23, p = 0.732) for any of the nine tests. When all nine 6-second Wingate tests are analyzed, the test had an ICC of 0.95 (0.92–0.97) and a SEM of 40.0 Watts (SEM% = 3.7%, 95% CI = 3.4% - 4.4%). Table 2 displays the reliability values of the best or average MPP values on each day. Absolute reliability improved when using the best MPP from each day (SEM = 31.3W, SEM% = 2.9%) or based on the average MPP value of each day (SEM = 29.1W, SEM% = 2.7%). Bland-Altman plots for inter-day comparisons between the best MPP for pairs of days (baseline vs day 1, day 1 vs day 2, and day 2 vs day 3) is presented on Fig 2. The SEM was lower than the SWC when the best (SEM = 31.3W vs SWC = 39.7W) or average (SEM = 29.1W vs SWC = 37.6W) MPP values from each day are compared.

**Table 2:** Characteristics and reliability measures of the best and the average peak power test on each day.

	Baseline	Day 1	Day 2	Day 3	P-value	ICC	SEM	SEM%	SWC
Best MPP (Watts)	1082.8 ± 192.3	1074.9 ± 186.9	1075.5 ± 202.4	1088.7 ± 207.4	.32	0.97 (0.95–0.99)	31.3W	2.9 (2.7%– 3.1%)	39.7
Average MPP (Watts)	1051.0 ± 179.1	1056.7 ± 180.4	1057.1 ± 193.5	1073.3 ± 208.8*	.07	0.97 (.95 - .98)	29.1W	2.7 (2.5% - 2.9%)	37.6

* Significantly different than day 2 (p < .05).

Data for MPP values are presented as mean ± standard deviation. Values in parentheses represent 95% CI. MPP: Maximal peak power; ICC: Intraclass correlation coefficient; SEM: Standard error of measurement; SEM%: Standard error of measurement as a percentage; SWC: Smallest worthwhile change.

Table 3 provides inter-day reliability values across the testing days. Relative reliability was similar across days (ICC values of 0.95 to 0.97), but absolute reliability (SEM% of 3.7% to 2.9%) improved throughout the study, when analyzing the results of all testing days to when just the last two testing days are compared.

**Table 3:** Changes in inter-day reliability of MPP values across testing days.

	Mean MPP (Watts)	P-value	ICC	SEM	SEM%	SWC
BL, D1, D2, D3	1058.5 ± 184.2	.11	0.95 (0.92–0.97)	40.0W (947.6–1169.3W)	3.7% (3.4%– 4.4%)	37.7W
D1, D2, D3	1062.3 ± 193.3	.06	0.97 (0.94–0.98)	34.0W (968.0–1156.5W)	3.2% (2.9% - 3.5%)	38.6W
D2, D3	1065.2 ± 199.1	.06	0.97 (0.95–0.99)	31.6W (977.6–1152.7W)	2.9% (2.7% - 3.2%)	40.0W

Data for MPP values presented as mean ± standard deviation. Values in parentheses represent the 95% CI. BL: Baseline; D1: Day 1; D2: Day 2; D3: Day 3; ICC: intraclass correlation coefficient; SEM: Standard error of measurement; SEM%: Standard error of measurement as a percentage; SWC: Smallest worthwhile change.

### Reliability and MPP values from the 1^st^ and 2^nd^ 6-second Wingate test across days

Reliability values for days 1 to 3 for the first and second 6-second Wingate test are presented on Table 4. There was a significant main effect of time for the second 6-second Wingate test (F(2, 52) = 3.59, p = .035, partial η^2^ = .121), with a significant difference between days 2 and 3 (mean difference 22.2W, p = .017, 95% CI = 3.3 to 41.1W). There was no statistically significant difference between the average MPP values from the first and second trials across days (1058.4 ± 196.6 vs 1066.2 ± 191.8W, p = .22). Both the 1^st^ and 2^nd^ 6-second Wingate test presented lower SEM than the SWC (SEM = 34.6W vs SWC = 39.3W, and SEM = 30.2W vs SWC = 38.2W, for the first and second trials on each day, respectively).

**Table 4:** Inter-day reliability values for the first and the second MPP trials on days 1 to 3.

Trial	Day 1 (Watts)	Day 2 (Watts)	Day 3 (Watts)	Mean MPP (Watts)	P-value	ICC	SEM (Watts)	SEM%	SWC (Watts)
1^st^ 6-second Wingate	1049.0 ± 180.8	1058.1 ± 201.1	1068.2 ± 213.0	1058.4 ± 196.6	.35	0.96 (0.94–0.98)	34.6	3.2% (3.0%– 3.6%)	39.3
2^nd^ 6-Second Wingate	1064.3 ± 183.5	1056.1 ± 190.0	1078.3 ± 206.3*	1066.2 ± 191.8	.035	0.97 (0.95–0.98)	30.2	2.8% (2.6% - 3.1%	38.2

* Significantly different than day 2 (p < .05).

Data for MPP values presented as mean ± standard deviation. Values in parentheses represent the 95% CI. MPP: Maximal peak power; ICC: Intraclass correlation coefficient; SEM: Standard error of measurement; SEM%: Standard error of measurement as a percentage; SWC: Smallest worthwhile change.

## Discussion

The primary aim of this study was to determine if a 6-second Wingate test could be utilized to reliably determine MPP in a cohort of trained endurance athletes. The main findings showed that: (1) a 6-second Wingate test provides a reliable estimate of MPP in trained endurance athletes habituated to cycling, (2) a learning effect did not occur in this group of athletes, and (3) there was no difference in MPP values between the first and second trial on each testing day.

### Inter-day reliability

When all nine trials are considered, the test shows excellent relative reliability (ICC = 0.95, 95% CI = 0.92–0.97), with no systematic differences across the trials in MPP values or time to peak power. The test also demonstrated excellent (SEM% < 5%) absolute reliability with a low standard error of measurement (SEM = 40.0W, SEM = 3.7%, 95% CI = 3.4% to 4.4%). While relative reliability was similar throughout the study, absolute reliability improved when the average (SEM% = 2.7%, 95% CI = 2.5% - 2.9%) or best (SEM% = 2.9%, 95% CI = 2.7% - 3.1%) MPP values on each day are used, corroborating previous recommendations that when multiple trials are performed, using the average of the results leads to better reliability values [29].

While the differences in protocols, participants’ training status and cycling experience makes comparisons between studies difficult, the current results demonstrate that a 6-second Wingate test shows similar reliability values as other 6-second tests that have been previously utilized to determine MPP, with reliability values in the literature ranging between 3.0 to 6.0% for SEM% and between 0.80 to 0.97 for ICC [17,18,20]. The one other study that utilized a 6-second Wingate test to determine MPP [18], reported similar ICC (0.93 to 0.94) and SEM% (3.5% to 5.1%) to the values seen in this study (ICC = 0.95 and SEM% = 3.7%), despite recruiting physically active, but untrained participants. However, Kavailauskas and Phillips (2016) determined that the test was not sensitive to detect a meaningful change in the participants’ MPP, with the SEM being larger than the SWC on all testing days. In this study, the SEM was smaller than the SWC when the values for days 1 to 3 are compared. It is possible that the cycling experience of the participants in this study allowed them to better replicate their performance across trials, positively impacting the test’s sensitivity. While caution is advised when interpreting the changes from the first day of testing (baseline), it must be noted that performing two trials and utilizing either the average or the best MPP values from this day eliminates the issue, with both methods demonstrating a good sensitivity to detect a true change in the participant’s MPP across all testing days in this study.

Throughout the study, absolute reliability values gradually increased (Table 2). The changes (approximately 0.8% for the SEM%, from all tests to days 2 and 3 only) are smaller than what has been reported in other studies. Mendez-Villanueva et al. (2007) showed that the SEM% improved from 4.4% across all trials to 1.8% when only the last two days of testing (out of 4) were considered. A similar effect was reported by Kavaliauskas and Phillips (2016), with a large reduction in the SEM% from 6.0% to 4.1% across the 4 trials, although these changes were only reported in female participants. However, these studies utilized participants that were untrained, but physically active [18] or active and involved in a range of sports, but with no experience in cycling [20]. As the ability to produce maximal cycling efforts is influenced by the participant’s cycling skills [30], the cycling experience from the participants in this study is also likely to explain these findings, as it might have allowed them to produce more consistent efforts across all trials, with a further improvement in their ability to perform these efforts after multiple tests.

### Intra-day reliability

The within-day reliability of the test can also be considered excellent (Table 4). To the authors’ knowledge, only one study [19] has analyzed the within-day reliability of a 6-second all-out effort in trained cyclists. The study had participants complete 10 x 6-second all-out efforts with a fixed cadence, with the results showing higher ICC (0.96 to 0.98) and lower %SEM values (2.19%) compared to the ones in the current study. The use of a fixed cadence has been hypothesized to lead to a better standardization of the test, and consequently, more reliable results than protocols with a flying start, which could explain the difference in the results. However, the fact that the within-day reliability values on day 3 (ICC of 0.98 and SEM% of 2.4%) are similar to those reported by Fernandez-Pena et al. (2007) indicates that when trained endurance athletes perform multiple 6-second all-out efforts, a flying start, aiming for a maximal cadence, can produce equally strong reliability values as a protocol with a fixed cadence. Lastly, except at baseline, the SWC from each testing day demonstrated that in this cohort, a 6-second Wingate test can be utilized to detect a meaningful change in the participant’s MPP, with the SEM being the same (day 2) or smaller than the SEM (days 1 and 3).

### Learning effect

The lack of differences in MPP values across days shows that a learning effect did not occur. A learning effect is characterized by a significant improvement in performance in the first few trials of a novel task [29]. Previous studies that utilized a 6-second all-out effort to determine MPP have reported such effect in trained non-endurance athletes that were unfamiliar with cycling [20], and in physically active, but untrained individuals [13], with the reported improvement ranging between 5% and 10%. However, when participants are familiar with cycling [22] or with the performance of short sprints [17], no learning effect is evident. In this study, the magnitude of performance changes between the first two trials at baseline (15.4 watts or approximately 1.5%), along with the lack of systematic differences across any of the trials, indicates that a learning effect did not occur, corroborating what has been previously reported in trained endurance cyclists [22]. However, it must be noted that the first trial of the baseline testing day likely worked as a familiarization trial for most participants. The trial had the lowest MPP values across all trials in the study (1042.0 ± 190.5W) and 4 of the 8 documented failed tests. The results obtained during the first 6-second Wingate trial at baseline also contributed to the lower reliability values reported for this day. For example, when only trials 2 and 3 during baseline are considered, ICC values improve from 0.95 to 0.97, and the SEM% is reduced from 3.7% to 3.0%. Therefore, it is advised that researchers and practitioners interpret the results of the first 6-second Wingate test with caution, particularly if the athletes are not habituated to this type of testing. In this case, performing two all-out efforts during baseline is recommended to reliably determine the athlete’s MPP (with the highest MPP value attained providing the best indication of the athlete’s MPP).

### Differences in MPP values and reliability between the 1^st^ and 2^nd^ trials across days 1 to 3

There were no differences in the MPP values obtained from the first or the second trials on days 1 to 3. Previous studies have shown that 70% of participants achieve their best MPP value during the second trial of the day [17], or that the fourth trial of the day produces the best MPP results [19], highlighting the importance of performing multiple trials within the same day to determine the participants’ MPP. The performance of multiple trials to determine MPP is also common in field- and laboratory-based endurance studies [4,5,21]. Our results, however, indicate that this might not be necessary. In this study, 11 participants (approx. 40%) had higher MPP values on the first all-out effort of the day on day 1. On days 2 and 3, 13 out of 27 participants (approx. 48%) achieved their best result in the first trial. Both the 1^st^ and 2^nd^ trials also demonstrated similar reliability, with marginally better values on the 2^nd^ 6-second Wingate across days (ICC of 0.96 vs 0.97, and SEM% of 3.2% vs 2.8%, for the 1^st^ and 2^nd^ trials, respectively). In addition, there was no difference in the average of the MPP values from the first (1058.4 ± 196.6W) or second (1066.2 ± 191.8W) trials across days. Considering the lack of differences between the first and second trials and the similar reliability values, our results demonstrate that a single all-out effort is likely sufficient to reliably establish MPP in trained endurance athletes, particularly as the athletes become more familiar with the test.

### Potential for multiple trials to enhance MPP

It is noteworthy that the 4^th^ day of testing had the two highest MPP values of the study, with the average MPP values showing a statistically significant difference from day 2 (1057.1 ± 193.5 vs 1073.3 ± 208.8, for days 2 and 3, respectively). As a learning effect is unlikely to explain these results, a possible explanation for these improvements in MPP (approximately 1.75%) is that a training effect occurred. Previous research has shown that trained road cyclists had a 5.6% improvement in MPP following 10 to 12 sessions (performed over 6 weeks) of sprint training (12 sprints of 4 to 8 seconds per session) [31]. These improvements were attributed to the specificity of the training efforts and the potential effect of repeating a novel stimulus. Indeed, short efforts at a high pedalling rate have been shown to improve power output under similar conditions, likely due to improved neuromuscular coordination [30], which might influence the individual’s rate of force development [32]. These factors could explain the improvement seen in this study in a cohort of athletes that was not used to performing all-out efforts, with 21 of the 27 participants achieving a higher average MPP value on day 3. Since there were no changes in the participants’ time to peak power on day 3 compared to other days, an improvement in skill when performing all-out efforts is unlikely to explain these changes. As such, these results could indicate a neuromuscular adaptation to short, all-out sprints. Future studies are required to elucidate the volume of training (frequency and number of all-out efforts) that is required for a change in MPP to occur. This is of interest to researchers to understand if future study designs need to consider the volume of all-out efforts performed by the participants to ensure that the results are not confounded by a training effect. Given the increased importance of performing repeated, high-intensity efforts during endurance events [4,9] and the importance of MPP and APR in these scenarios [2,5,11], these studies could also help to determine the time-course of adaptation to all-out efforts, assisting coaches and sport scientists in deciding when this type of training should be included prior to a competition.

In addition to showing that a 6-second Wingate test is a reliable test to determine MPP in trained endurance athletes, the results of this study also add three important contributions to the current literature. First, considering that the MPP values at baseline were similar to the values seen in the other testing days, it indicates that a prior maximal incremental test does not lead to a significant level of neuromuscular fatigue that would inhibit the athlete’s ability to produce their maximal peak power, as previously shown [33,34]. This is of interest to researchers and sport scientists as it demonstrates that a 6-second all-out test can provide reliable results even if performed in the same session as other tests or maximal aerobic efforts, potentially reducing the number of testing days an athlete might be required to perform. Second, it shows that for trained endurance athletes, a rolling start, where participants are instructed to reach their highest cadence prior to the load being applied, leads to reliability values that are similar to those seen in other studies with a static start [17,20], a fixed cadence [19], or to studies where the load is applied at a lower cadence [18]. Such protocols were deemed to lead to a better standardization of the test, and thus, better reliability values [23,35]. Our results demonstrate that trained endurance athletes can reliably repeat all-out efforts when asked to produce their maximal cadence. This is relevant as a “flying” start is commonly used in field-based endurance studies [5]. This also demonstrates the reliability of the Velotron ergometer to determine MPP during a 6-second Wingate test, adding to the existing literature on the topic [25]. Lastly, our results corroborate the notion that a rolling start leads to a shorter time to peak power [35]. In this study, MPP was achieved, on average, in 0.66 seconds, compared to a TTP of approximately 1.8 seconds during a 6- or 30-second Wingate test [14,18]. This provides further support to the notion that even shorter tests (for example, 3 to 4 seconds) might be sufficient to determine MPP in trained endurance athletes, as previously demonstrated [22]. While the metabolic demands of a 3-second all-out effort are quite low [36], these demands increase significantly with longer efforts, with a 3-fold increase in oxygen consumption and a 7-fold increase in blood lactate accumulation when compared to an 8-second all-out effort [37], for example. In this context, a shorter effort could reduce the physiological demands of the test and increase its applicability, as the test could easily be performed as part of a regular training session or a battery of tests.

The fact that the testing days in this study were separated by only 48–72 hours of rest could limit the applicability of the results. Fatigue from the previous testing days or a lack of motivation could potentially explain the fact that day 2 had similar reliability values as baseline, when other studies in the literature [20] show a gradual improvement in performance across testing days. In addition, nutrition, sleep, and the participants’ own training were not controlled for throughout the study, despite all participants confirming that they followed the researchers’ instructions to avoid strenuous exercise and ensure the consumption of a similar meal prior to each testing day. Data collection occurred during an 8-month period with some participants at different phases in their training (e.g., general preparatory phase, specific phase, competitive season). It is likely that as their training became more specific, some participants would include short, maximal sprints as part of their training, or would perform similar efforts during competition. The reliability of an all-out test to determine MPP in trained endurance athletes who regularly perform these types of efforts remains to be determined.

## Conclusions

The results of this study demonstrate that a 6-second Wingate test can be used to reliably determine maximal peak power in trained endurance athletes. The test showed no systematic differences in MPP values across all trials and excellent reliability values between and within days. There was also no significant difference between the MPP values obtained on the first or second trial on each day (with the 2^nd^ Wingate showing an average improvement of 0.7%). If participants are unfamiliar with the performance of all-out sprints with a "flying” start, two trials are recommended on the first day of testing to determine their MPP. Subsequently, a single 6-second all-out effort is likely sufficient to reliably determine MPP in trained endurance athletes. Future studies should investigate if shorter tests (for example, a 3-second all out effort) can produce similar results. Given the potential improvement in MPP in as few as 4 testing days in this study, further studies are required to understand how many efforts need to be performed for improvements in this characteristic.

## Supporting information

S1 File Data for each trial in the study. (XLSX)
